# Levothyroxine Augmentation in Clozapine Resistant Schizophrenia: A Case Report and Review

**DOI:** 10.1155/2015/678040

**Published:** 2015-05-11

**Authors:** Ruohollah Seddigh, Somayeh Azarnik, Amir-Abbas Keshavarz-Akhlaghi

**Affiliations:** ^1^Mental Health Research Center, Iran University of Medical Sciences, Tehran, Iran; ^2^Shahid Rajaie Cardiovascular, Medical and Research Center, Iran University of Medical Sciences, Tehran, Iran

## Abstract

There are many reports that show different thyroid abnormalities in schizophrenia without clear establishment of their role in etiology and treatment outcome of schizophrenia. Among these reports, there are only a few that consider a role for thyroid hormones as augmenting agents in the treatment with antipsychotic drugs. This case report outlines symptom subsidence of a patient with clozapine refractory paranoid schizophrenia and normal thyroid function who added levothyroxine to clozapine and found that symptoms of psychosis returned once levothyroxine was discontinued. Although this observation needs to be confirmed in controlled clinical trials, we aimed to discuss possible hypothesized mechanisms underlying this observation.

## 1. Introduction

Although its clinical implication still remains disputable, psychiatrists often use thyroid hormones to augment antidepressant effects in treatment-resistant major depressive disorder [1]. However, in psychotic disorders, the issue of using thyroid hormones as augments to antipsychotic drug use is associated with more ambiguities. Studies indicate that thyroid hormones have a role in normal development of the central nervous system [2], neuronal myelination [3], and proinflammatory responses in the brain, as well as in regulating dopaminergic, serotonergic, glutamatergic, and GABAergic systems [4], in synthesis and regulation of brain receptors, and also in response to treatment [5]. Thyroid hormones can also play a role as neurotransmitter [4]. Furthermore, in clinical terms, there is considerable overlap between cognitive aspects of hypothyroidism and negative symptoms of schizophrenia [3]. Thus, it may be possible to attribute an etiological or even therapeutic role to thyroid hormones in schizophrenia. Regarding treatment strategies, there are only a few reports that consider a role for thyroid hormones as augmenting agents in the treatment with antipsychotic drugs [6–8].

About 20–30% of patients with schizophrenia do not properly respond to antipsychotics [9]. In such cases, one of the effective options is treatment with clozapine, which is supported by a huge body of evidence. However, 50–70% of recipients do not adequately respond to clozapine either [10, 11]. For this group of patients, various augmentation strategies have been proposed, from a variety of psychotherapies to several medications and electroconvulsive therapy (ECT). However, due to a paucity of controlled trials, strong evidence-based guidelines cannot be provided [12]. Thus, clozapine refractory cases could be a challenge for clinicians. The case presented here emphasizes the possible role of thyroid hormones as augmentation strategies for patients with clozapine refractory schizophrenia.

## 2. Case Presentation

A 25-year-old unemployed single woman with refractory schizophrenia was referred to the university psychiatric clinic. Her problem had begun at the age of 20 years with social withdrawal and loss of self-care. Six months after the first signs of illness, she was experiencing clear auditory hallucinations, paranoid delusions, and significant deterioration of functions, both socially and individually. The patient had no previous history of medical or psychiatric disorders and had never used alcohol or drugs. She had been hospitalized twice (at the age of 23 and 24 years), and, at both times, the patient had been treated with antipsychotic drugs, including risperidone (6 mg/day for three months), haloperidol (20 mg/day for four months), olanzapine (20 mg/day for two months), perphenazine (16 mg/day for three months), and aripiprazole (20 mg/day for three months), but her psychosis never diminished in spite of good compliance and some medication side effects.

In psychiatric examinations, the patient's major symptoms included anxiety, auditory and visual hallucinations, persecutory and reference delusions, verbal aggression, irritability, insomnia, social withdrawal, and clear functional failure. The patient thought her family was going to harm her, for example, by trying to poison her food and by chasing her when she was not with the family. The patient also heard voices saying that the family was going to harm her. Visual hallucinations included seeing vague scenes. Secondary to the delusions, she had experienced great fear and anxiety and sometimes became aggressive and impulsive to defend herself. The patient did not have any insight into the unreal nature of these experiences. When evaluated with the Positive and Negative Syndrome Scale (PANSS) [13], scores were as follows: positive scale = 31, negative scale = 25, and general psychopathology = 70. Her paraclinical examinations, complete blood count, thyroid tests, kidney and liver function tests, addiction tests (including amphetamine, cannabis, opium, and alcohol), brain magnetic resonance imaging (MRI), and electroencephalography (EEG) were all within reference limits. Evaluation of the medical and psychiatric family history of the patient did not show any illness among relatives with the exception of hypothyroidism in the mother.

The patient was hospitalized with a confirmed diagnosis of refractory paranoid schizophrenia, and clozapine was administered with a gradually increasing dose, up to 600 mg per day. After six weeks, she was significantly calmer with lower anxiety and aggression, her sleep quality had improved, and her appetite had increased. However, she was still experiencing social withdrawal, persecutory and reference delusions, and visual hallucinations. Despite good drug adherence, psychosis continued four months after discharge. Afterwards, without our knowledge, the mother, who had hypothyroidism, advised the patient to use levothyroxine (at a dose of 0.1 mg per day) in addition to clozapine. After two weeks with this treatment, hallucinations and delusions completely subsided, and the patient's social relations improved. When we were informed, discontinuation of levothyroxine was advised, given her normal thyroid tests. About three weeks after the removal of levothyroxine, psychosis symptoms gradually reappeared (despite still taking clozapine). The patient was again experiencing hallucinations and delusions.

Thyroid tests were once again requested, with the following results (reference ranges included in parenthesis): thyroid stimulating hormone (TSH) = 3.5 (0.2–4.3) *μ*IU/mL, free thyroxine (T4) = 1 (0.7–1.25) ng/dL, total T4 = 8.6 (5.1–14.1) *μ*g/dL, free triiodothyronine (T3) = 3.5 (2.4–4.2) pg/mL, total T3 = 1 (0.5–2.2) ng/mL, and anti-thyroid peroxidase antibody = 23 (<35) IU/mL. The patient's thyroid status was also reported as normal in endocrinology consultation. Considering the normal test results, ECT was prescribed for the patient. However, instead of ECT, the patient again added levothyroxine (0.1 mg/day) to her clozapine (600 mg/day) without our knowledge. Symptoms of psychosis disappeared after two weeks and did not recur during one year of follow-up. Also the thyroid tests six and twelve months after restarting medication with levothyroxine were within the reference ranges (in the 6th month, TSH = 1 (0.2–4.3) *μ*IU/mL, total T4 = 10.6 (5.1–14.1) *μ*g/dL, and total T3 = 0.8 (0.5–2.2) ng/mL (0.5–2.2); in the 12th month, TSH = 1.4 (0.2–4.3) *μ*IU/mL, total T4 = 9.5 (5.1–14.1) *μ*g/dL, and total T3 = 1.3 (0.5–2.2) ng/mL).

## 3. Discussion

About 30–36% of patients with chronic schizophrenia have abnormal thyroid tests, but, in clinical terms, they are euthyroid [14, 15]. These abnormalities may disappear following successful treatment of schizophrenia and may also have a correlation with treatment response to antipsychotics [16]. For instance, it has been observed that higher T3 serum levels in patients with chronic schizophrenia are related to their better cognitive functions and lower extrapyramidal drug side effects [17]. It has also been seen that high basal TSH is associated with poorer response and blunted TSH response to thyrotropin releasing hormone (TRH) and a high level of T4 before treatment with better response to treatment [18]. T4 levels before treatment are also positively correlated with severity of the disorder [16]. Although the results of studies are contradictory, they mostly cite increased total and free T4 in patients with schizophrenia before treatment and their normalization after treatment [4].

Although the above arguments do not explain the effects of levothyroxine on the patient described here, this observation can perhaps be explained by less supported findings. For example, it has been observed that the prevalence of anti-thyroid antibodies is higher in patients with chronic schizophrenia than in the healthy population [19], and signs of thyroid and pituitary deterioration can also be observed in autopsies [20]. Moreover, there is a mutual relationship between thyroid function and schizophrenia: on the one hand, dopaminergic hyperactivity in schizophrenia affects the pituitary gland, which leads to reduced TSH and therefore reduced thyroid hormones [21]. On the other hand, an altered thyroid status can change the sensitivity of dopamine receptors and thus change their response to antipsychotics [22]. Specifically, there is a report indicating that clozapine, olanzapine, and quetiapine can change levels of thyroid hormones and occasionally lead to clinical or subclinical hypothyroidism, which in some cases requires treatment [23]. Furthermore, in patients under treatment with antipsychotics, even in the absence of abnormal thyroid hormone blood level, deregulated deiodinase activities, as well as N-glucuronidation of thyroid hormones, and, as a consequence, alterations in thyroid hormones level may be observed [4]. All of the above arguments need to be replicated with more precise methods.

According to our inquiry, treatment augmentation with thyroid hormones has rarely been reported in schizophrenia: a case of risperidone augmentation with triiodothyronine [7], a case of chlorpromazine augmentation with triiodothyronine [6], and a case of weekly administration of levothyroxine that led to increased patient compliance [8]. However, use of thyroid hormones can be seen among old treatments for schizophrenia, before the age of antipsychotics, with occasional high doses (especially T3), without hyperthyroidism as a side effect. Advocates of such treatments believed that patients with schizophrenia had abnormal resistance to thyroid hormones, which was probably due to peripheral insensitivity to thyroid hormones or presence of thyroid receptor antagonists in the body [24]. According to these observations, it can be argued that, despite a normal thyroid profile, our patient probably did not have a favorable thyroid function (for various reasons), which had led to an inadequate response to clozapine. Nevertheless, this explanation is only hypothetical, based on the few reports that exist in this area, and requires further accurate studies.

Finally, there are reports that show positive effect of levothyroxine augmentation in refractory bipolar disorder, specifically in its rapid cycling form [25] and in bipolar depression [26], and especially in supratherapeutic doses [25, 27]. These findings might suggest further evidence for the probable role of thyroid hormones in managing the treatment of refractory schizophrenia, because of the genetic correlation between bipolar disorder and schizophrenia [28].

A limitation of this study is the lack of serum concentrations of clozapine. However, according to the patient's behavior at the clinical ward and the reports from herself and her family, it seems likely that the patient took the prescribed medications during the whole time frame described above. Still, serum concentrations of the antipsychotic compounds could have given a clue as to whether the antipsychotic medications were nonsufficient in the doses prescribed.

## Abbreviations

PANSS: Positive and Negative Syndrome Scale
MRI: Magnetic resonance imaging
EEG: Electroencephalography
TSH: Thyroid stimulating hormone
ECT: Electroconvulsive therapy
GABA: Gamma aminobutyric acid.
